# Actin, connexin-43 and GAP-43 expression in gliomas: real-world associations with IDH status, tumor burden and survival

**DOI:** 10.1007/s11060-026-05745-7

**Published:** 2026-08-08

**Authors:** Aleksandrs Krigers, Matthias Demetz, Lisa Bergmeister, Patrizia Moser, Adelheid Woehrer, Claudius Thomé, Christian F. Freyschlag

**Affiliations:** 1 Department of Neurosurgery, Medical University of Innsbruck, Anichstr. 35, Innsbruck, AT-6020 Austria; 2 Institute of Pathology, Neuropathology & Molecular Pathology, Medical University of Innsbruck, Innsbruck, Austria

**Keywords:** Glioma, Connexin-43, Actin, Gap-junctions, Survival

## Abstract

**Background:**

Connexin-43 (Cx43), GAP-43 and actin are involved in cellular communication and cytoskeletal dynamics in gliomas, yet their clinical relevance remains largely unclear. We investigated their expression and association with survival and tumor characteristics in IDH-mutant and IDH-wildtype gliomas.

**Methods:**

134 adult patients operated for newly diagnosed gliomas WHO grades 2–4 in our center were included. Immunohistochemical expression of Cx43, GAP-43 and actin was assessed. Several clinical parameters, including progression-free (PFS) and overall survival (OS), were acquired from our institutional database.

**Results:**

In IDH-wildtype tumors, higher Cx43 expression was associated with prolonged OS (*p* = 0.032). Whereas IDH-mutant gliomas showed significantly lower expression of Cx43 (*p* = 0.029) compared to IDH-wildtype tumors. Actin expression correlated with prognostically unfavorable higher WHO grade (*p* < 0.001), IDH-wildtype status (*p* < 0.001), preoperative contrast enhancement in MRI (*p* = 0.009) and decreased functional status (*p* < 0.001). Consequently, stronger actin expression was significantly associated with shorter PFS (7.2 vs. 17.4 months, *p* < 0.001) and OS (17.1 vs. 81.5 months, *p* < 0.001). Incidentally diagnosed tumors exhibited reduced GAP-43 expression (*p* = 0.012). None of the proteins was associated with perioperative complications.

**Conclusions:**

Upregulated actin expression is strongly linked to more aggressive glioma behavior and reduced survival. Higher Cx43 expression could sustain an onco-protective role in prognostically unfavorable IDH-wildtype tumors. These findings provide clinically relevant translational evidence for the role of cytoskeletal and connectivity-associated proteins in glioma biology.

## Introduction

Gliomas are highly infiltrative primary brain tumors characterized by pronounced cellular plasticity and poorly understood interactions with the surrounding microenvironment [1–4]. These processes are closely linked to cytoskeletal dynamics, cellular connectivity, intercellular communication and neuron-to-glia interactions. Nevertheless, the underlying pattern of protein expression in human glioma tissue remains mostly unexplored [5–7].

Despite advances in molecular diagnostics, particularly the distinction between isocitrate dehydrogenase (IDH)-mutant and IDH-wildtype gliomas, tumor invasion and therapy resistance remain a major clinical challenge [8–10]. Hence, malignant gliomas are still incurable regardless of multimodal treatment strategies, which typically consist of surgical resection followed by radiotherapy and systemic chemotherapy [11]. Even with the best available therapy, survival remains poor, and tumors recur within a short time frame. Consequently, durable disease control is rare, and long-term survivors represent only a small fraction of affected individuals. Thus, there is a huge demand on a translational understanding of oncogenic pathways and recognizing novel specific prognostic and therapeutical targets.

Previous studies have suggested that Connexin-43 (Cx43), Growth-associated protein-43 (GAP-43) and actin play an important role in the functional neuro-oncological network by linking intercellular communication, cytoskeletal remodeling and neurite-like structural plasticity in glioma cells [6, 12–15]. This coordinated protein setup has been proposed to facilitate tumor cell integration into neural circuits, thereby promoting invasive growth patterns, adaptive connectivity and clinically important tumor behavior [13, 15]. Cx43 as the most abundant gap junction protein in the human central nervous system plays a pivotal role in cell-to-cell communication, migration and network formation [14, 16]. In gliomas, Cx43 has been implicated in tumor invasion, intercellular coupling and treatment resistance. Though, its exact role appears context-dependent and may vary across molecular subtypes [15, 17, 18]. GAP-43 is a well-established marker of neuronal growth cones and synaptic plasticity. It has recently gained attention in glioma biology due to its association with tumor cell motility, neurite-like outgrowth and interactions with neuronal structures [10, 19–21]. Actin, a fundamental component of the cytoskeleton, is essential for maintaining cellular shape, migration and invasion and represents a central effector of cytoskeletal remodeling in malignant cells [22–24].

While experimental studies suggest that Cx43, GAP-43 and actin are functionally interconnected and may jointly contribute to glioma cell plasticity and invasiveness, comprehensive analyses of their practical role in human glioma specimens are scarce [13, 15, 19]. Particularly, clinically relevant data comparing expression across IDH-mutant and IDH-wildtype gliomas in large, well-characterized patient cohorts with sufficient follow-up are limited. Thus, integrating translational laboratory findings with clinical outcome data allows a more comprehensive understanding of tumor behavior and helps to identify biologically relevant markers with practical prognostic and therapeutic implications.

In this study, we performed a large monocentric assessment of Cx43, GAP-43 and actin expression in tumor tissue of de novo diagnosed glioma patients. By systematical evaluation of these patterns and association with long-term clinical information, we aimed to depict real-world evidence of the connectivity-related features of gliomas and potential targets that could be used in future intercellular communication targeted treatment settings.

## Methods

### Patients and study cohort

Adult patients (≥ 18 years) with histologically confirmed gliomas of WHO grades 2–4, who were operated at our department, were screened. Both IDH-mutant and IDH-wildtype de novo glioma cases could be included. Patients’ clinical data were selected from a prospectively maintained neuro-oncological database and tissue acquired from the institutional biobank.

Routine indication for surgical intervention by suspected de novo glioma followed international treatment guidelines, with a primary aim of achieving maximal neurologically safe resection [25, 26]. It was defined as the largest possible tumor removal while preserving neurological function. The extent of resection was verified by early postoperative MRI [27]. Postoperative treatment strategies were discussed and individually determined in a multidisciplinary neuro-oncological tumor board according to standardized institutional practice [11, 26]. The postoperative course included routine neurological assessments, early imaging and structured long-term outpatient follow-up.

The neuro-oncological database and tissue bank are approved by the local ethics committee (AN5220 329/4.4), and written informed consent was obtained prior to surgery from all patients. The anonymized datasets generated and analyzed during the current study are available from the corresponding author upon reasonable request, subject to institutional and ethical regulations governing patient data.

### Clinical data, treatment characteristics and follow-up

Baseline functional status was assessed preoperatively using the Karnofsky Performance Score (KPS) and the clinical frailty scale (CFS). The KPS is a widely used performance scale where higher score indicates better functional independence and ability to carry out daily activities [28]. The CFS is a 9-point scale designed to evaluate overall frailty and vulnerability, incorporating functional status, comorbidities and level of dependence in daily life. Higher scores reflect greater frailty and reduced physiological reserve [29]. Together, these measures provide clinically complementary information regarding patients’ baseline functional capacity and resilience prior to surgical intervention. Presenting clinical symptoms at diagnosis, including seizures, focal neurological deficits, cognitive impairment and incidental findings, were systematically documented. Surgical variables included the extent of resection, which was categorized based on early postoperative magnetic resonance imaging. Information on administered adjuvant treatment was collected for all patients.

Longitudinal follow-up data comprised routine clinical assessments and serial neuroimaging. Tumor progression was defined radiologically according to the RANO criteria [27]. Progression-free survival (PFS) was calculated from the date of initial surgery to the date of radiological progression. Overall survival (OS) was defined as the interval between the date of first surgery and death from any cause. Patients without documented progression or death were censored at the time of last available evaluation.

### Routine histopathological and molecular evaluation

Following macroscopic inspection, tumor tissue was processed as formalin-fixed paraffin-embedded (FFPE) material and analyzed in routine diagnostic workflow by experienced neuropathologists. Tumor type and tissue representability were confirmed using hematoxylin–eosin (H&E) staining. Histopathological grading followed the World Health Organization (WHO) classification of tumors of the central nervous system, applying the most up-to-date edition available at the time of initial diagnosis. Subsequently, all tumor specimens were comprehensively reviewed and reclassified according to the 2021 WHO classification to ensure uniformity across the cohort [8, 30].

IDH1 R132H mutation status was assessed by immunohistochemistry (IHC). In patients younger than 40 years without immunohistochemical evidence of IDH1 mutation, additional DNA sequencing was performed to confirm IDH status. The proliferation index was determined by calculating the percentage of MIB-1 (Ki-67) positive nuclei relative to all tumor cells within representative areas. ATRX, EGFR and p53 expression were evaluated by immunohistochemistry as part of the routine workup.

### Immunohistochemistry for GAP-43, Connexin-43 and actin

Immunohistochemical staining for GAP-43, Cx43 and actin was performed using an automated staining system and commercially available antibodies (anti-GAP-43 EP890Y, 1:1000; anti-Cx43 P17302, 1:2000; anti-actin EPR16769, 1:4000; all Abcam) for FFPE samples. Appropriate positive and negative controls were included.

**Fig. 1:**
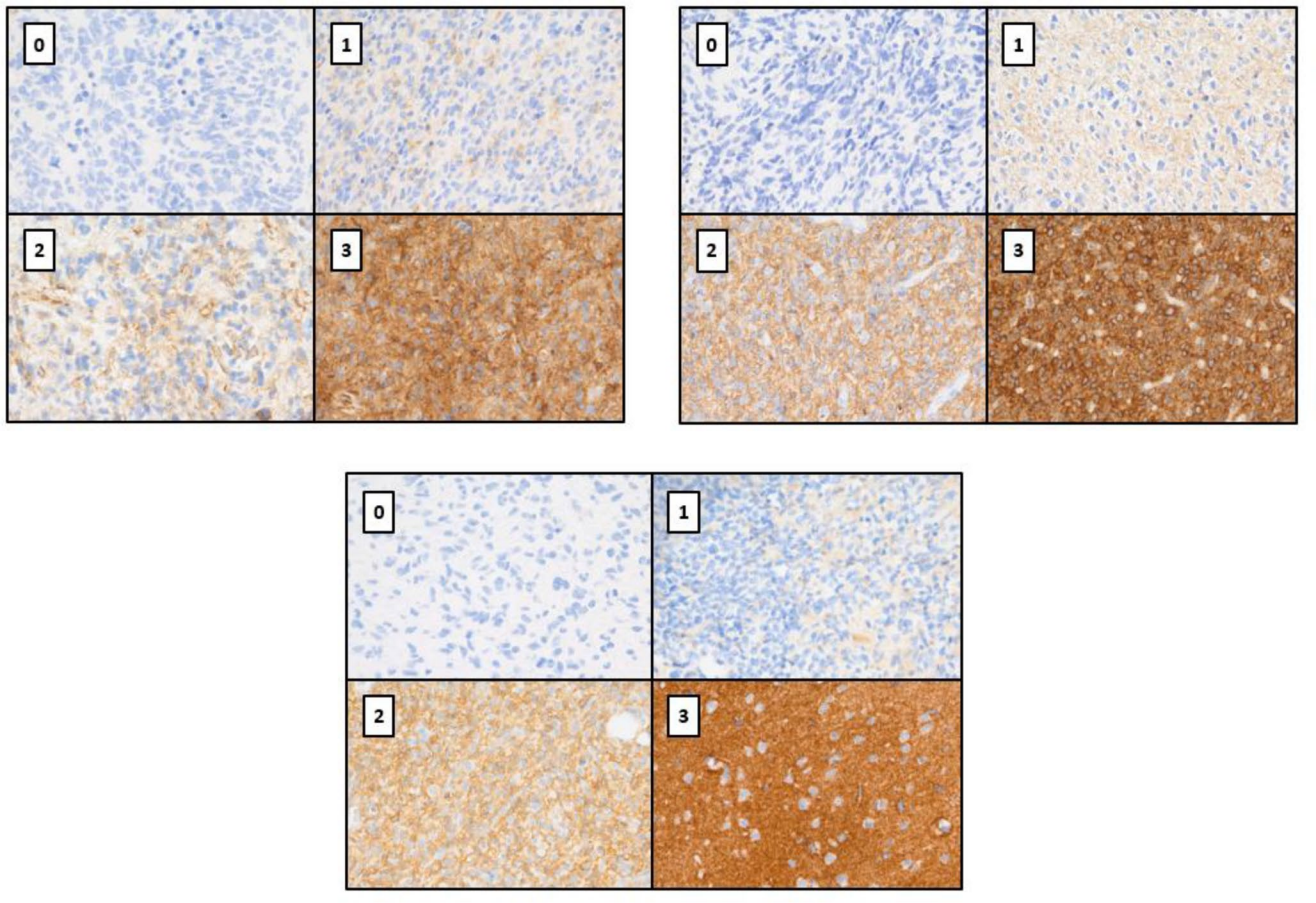
Representative immunohistochemical staining demonstrating expression of actin (top left), GAP-43 (top right), and Cx43 (bottom)

Protein expression of Cx43, GAP-43 and actin was assessed semi-quantitatively within glioma cell populations using a four-tiered scoring system: 0 (no expression), 1 (weak expression), 2 (moderate expression) and 3 (strong expression comparable to cortical tissue). Representative examples are provided in Fig. 1.

Consecutive H&E-stained sections were reviewed to confirm tumor localization and tissue adequacy. All samples were evaluated independently by two investigators experienced in neuropathology. In cases of discrepant scoring, the slides were reviewed jointly, and a consensus score was assigned according to routine neuropathological practice.

### Statistical analysis

Statistical processing was conducted using IBM SPSS Statistics for Mac OS (Version 30.0. Armonk, NY: IBM Corp.). Non-normally distributed scale and ordinal data were described with medians with interquartile ranges (IQR). Group comparisons and correlation analyses were performed using Mann–Whitney U tests, Chi-square tests and Spearman rank correlation, as appropriate. Survival was analyzed using Cox proportional hazards regression or Log-Rank test and visualized with Kaplan-Meier curves. To identify independent prognostic factors for PFS and OS, multivariable Cox proportional hazards regression analyses were performed including preoperative KPS, extent of resection, WHO grade, IDH status, and protein expression. Statistical significance was defined as a two-sided p value < 0.05 with a 95% confidence interval.

## Results

### Cohort description

We screened 183 patients who underwent surgical treatment an intracranial glioma at our institution. Forty-nine patients were excluded due to prior glioma treatment. Consequently, 134 patients were included in the final assessment.

The cohort consisted of 79 males (59%) and 55 females (41%). The median age at the time of surgery was 45 years (IQR 34–56). Median preoperative KPS was 90 (IQR 80–100) and the median preoperative CFS was 2 (IQR 1–3). A KPS of 90 reflects the ability to carry out normal activities with only minor symptoms, while a CFS score of 2 indicates that patients were generally well, independent and without significant functional limitations. Postoperatively, the median KPS remained at 90 (IQR 80–100), whereas the median CFS was 3 (IQR 2–4). At the first follow-up visit, functional status had recovered to baseline levels with median KPS of 90 (IQR 80–100) and median CFS of 2 (IQR 1–3).

Eloquent tumor location was present in 79 patients (59%). Eloquent tumor location refers to involvement of brain regions responsible for critical neurological functions such as language, motor control or sensory processing, where surgical intervention carries an increased risk of functional impairment. The most frequently affected anatomical region was the frontal lobe in 82 cases (61%), followed by the temporal lobe in 56 cases (42%).

Regarding clinical presentation, seizures were the leading presenting symptom and occurred in 79 patients (59%). Incidental tumor detection was documented in 11 cases (8%). Additionally, 15 patients (11%) had a history of a previous extracranial malignancy.

Considering surgical strategy, 122 patients (91%) underwent tumor resection as the initial procedure. Nine patients (6%) underwent biopsy only and three patients (2%) received a resection following an initial diagnostic biopsy. Adjuvant therapy was administered to 95 patients (71%). Among these, 82 patients (61% of the total cohort; 86% of treated patients) received concomitant radiochemotherapy according to the standardized Stupp protocol, which consists of fractionated external-beam radiotherapy administered together with daily temozolomide, followed by adjuvant temozolomide maintenance chemotherapy.

Preoperative MRI demonstrated a mean contrast-enhancing tumor volume of 8.9 cm³ (Standard error, SE 1.7) on T1-weighted sequences. Postoperatively, the mean residual contrast-enhancing volume was reduced to 0.3 cm³ (SE 0.09), corresponding to a mean extent of resection (EOR) of 96%.

Histopathological evaluation according to the 2021 WHO classification revealed CNS WHO grade 2 tumors in 34 patients (25%), CNS WHO grade 3 tumors in 27 patients (20%) and CNS WHO grade 4 tumors in 73 patients (55%).

IDH mutation was identified in 61 patients (45%), while 73 tumors (55%) were classified as IDH-wildtype. Among the available molecular data, 1p/19q codeletion was identified in 29 patients, while TERT promoter mutations were detected in 23 cases. Patients’ performance between IDH-mutated and IDH-wildtype gliomas can be found in Table 1. EGFR overexpression was detected in 62 tumors (46%), and ATRX was lost in 96 cases (72%).

The median follow-up time in our cohort was 46 months (IQR 33–59).

Patients’ performance and radiological data for IDH-mutated and IDH-wildtype gliomas can be found in Table 1.

**Table 1:** Median performance and mean radiological characteristics according to IDH status

	IDH-mutated	IDH-wildtype
Median KPS before surgery	90 (IQR 80–100)	90 (IQR 80–100)
Median CFS before surgery	2 (IQR 1–3)	3 (IQR 2–4)
Median KPS after surgery	90 (IQR 80–100)	80 (IQR 70–90)
Median CFS after surgery	2 (IQR 1–3)	3 (IQR 2–4)
Median KPS at first FU	90 (IQR 80–100)	90 (IQR 80–100)
Median CFS at first FU	2 (IQR 1–3)	3 (IQR 2–4)
Mean contrast enhancing tumor volume (cm^3^)	4.0 (SD 3.8)	13.0 (SD 12.4)
Mean extent of resection of CE tumor	99% (SD 3%)	93% (SD 9%)

### Cx43, GAP-43 and actin expression

**Fig. 2:**
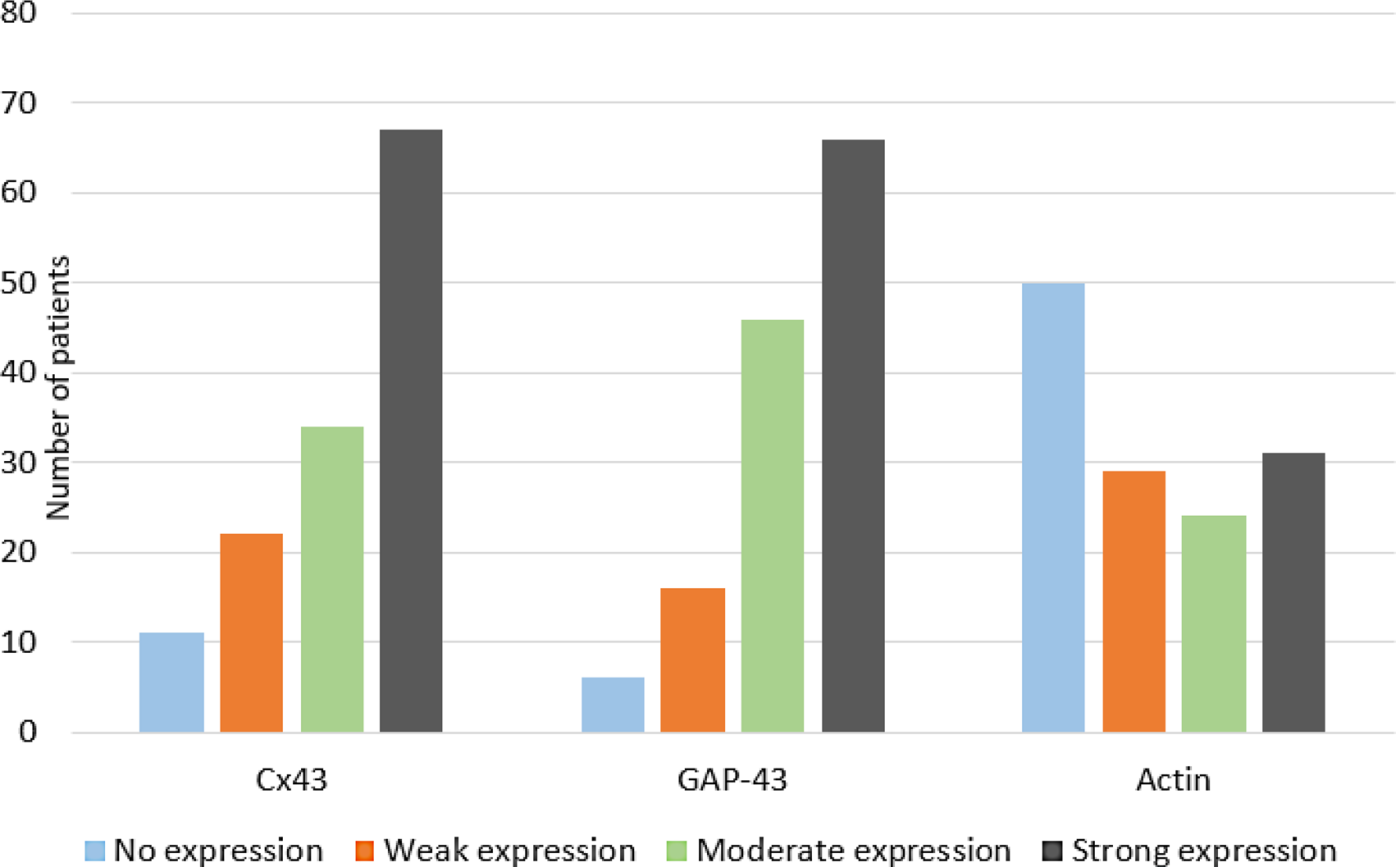
The distribution of immunohistochemical assessment of the expression levels of Cx43, GAP-43 and actin is provided with number of cases and per cent to entire cohort

The detailed distribution and semi-quantitative expression levels of Cx43, GAP-43 and actin are summarized in Fig. 2.

**Fig. 3:**
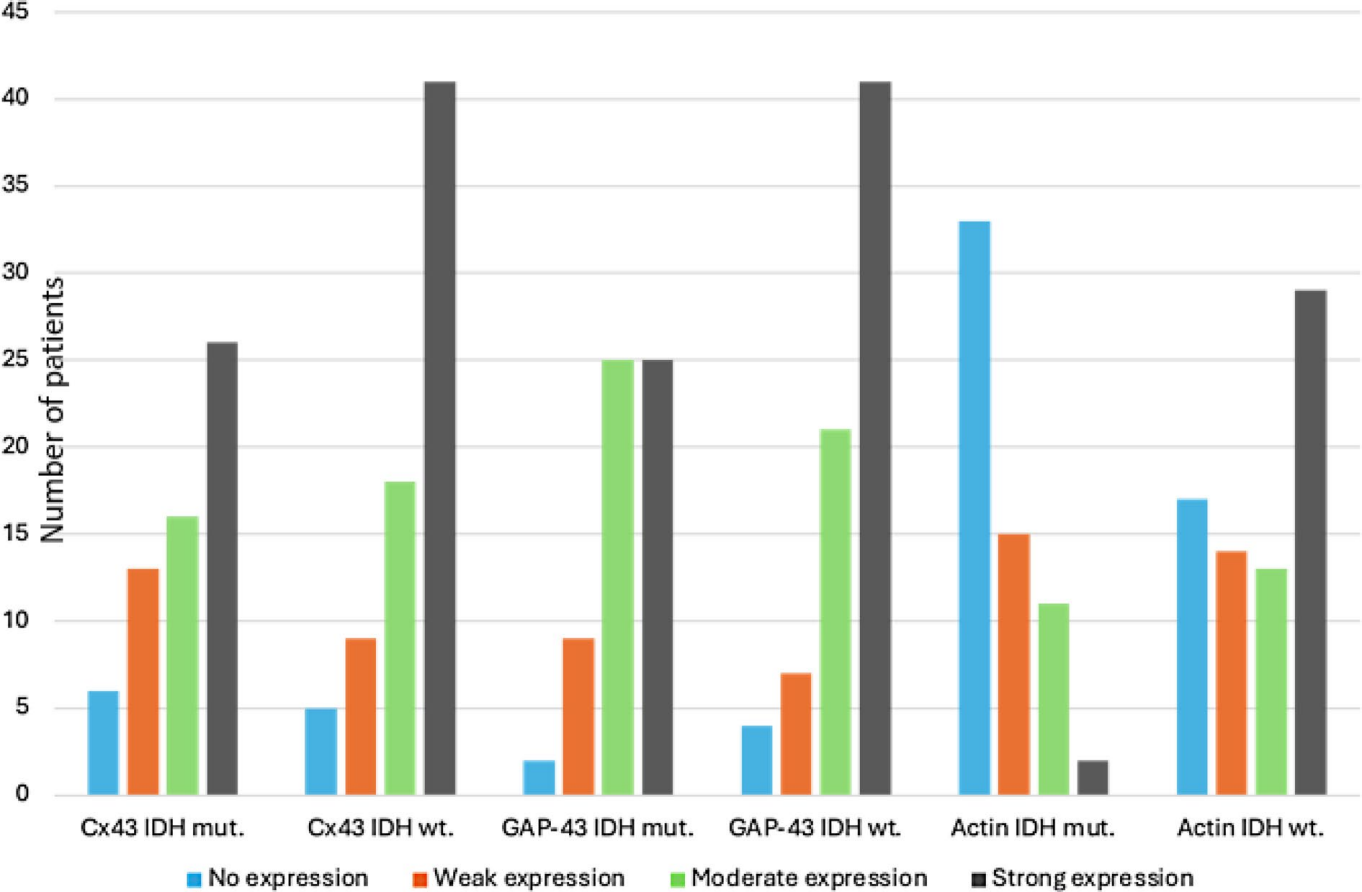
Bar graphics of the immunohistochemical assessment of the expression of Cx43, GAP-43 and actin in IDH-mutated and IDH-wildtype gliomas

Most gliomas demonstrated strong expression of Cx43 and GAP-43, whereas this pattern was not observed for actin, which showed a more heterogeneous and generally lower expression profile across cases. Expression of our three proteins in accordance with IDH-status is shown in Fig. 3.

### Association with clinical risk factors

Prognostically unfavorable IDH-wildtype tumors demonstrated significantly higher expression of actin (*p* < 0.001) and Cx43 (*p* = 0.029) compared to IDH-mutant tumors. Higher WHO grade was significantly associated with increased actin expression (*p* < 0.001), whereas no such relationship was identified for Cx43 or GAP-43. Asymptomatic patients with incidentally diagnosed tumors exhibited significantly lower GAP-43 expression (*p* = 0.012).

Higher actin expression was significantly associated with prognostically unfavorable volume of preoperative contrast enhancement on MRI (*p* = 0.009). Moreover, elevated actin expression correlated with poorer functional status, reflected by lower preoperative KPS and higher CFS scores (*p* < 0.001 each). These associations were not observed for Cx43 or GAP-43 expression.

None of our three proteins demonstrated a significant association with the occurrence of preoperative seizures. Similarly, expression levels of Cx43, GAP-43 and actin were not associated with the occurrence of postoperative complications, including new neurological deficits, hemorrhage or other surgical adverse events. No significant associations were observed between the expression levels of Cx43, GAP-43 or actin and patient gender, treatment modality, tumor location, or ATRX and EGFR status.

### Survival analysis

Median PFS in the entire cohort was 13.1 months (IQR 4.7–26.1) with a median OS of 46.4 months (IQR 13.3–82.5).

**Fig. 4:**
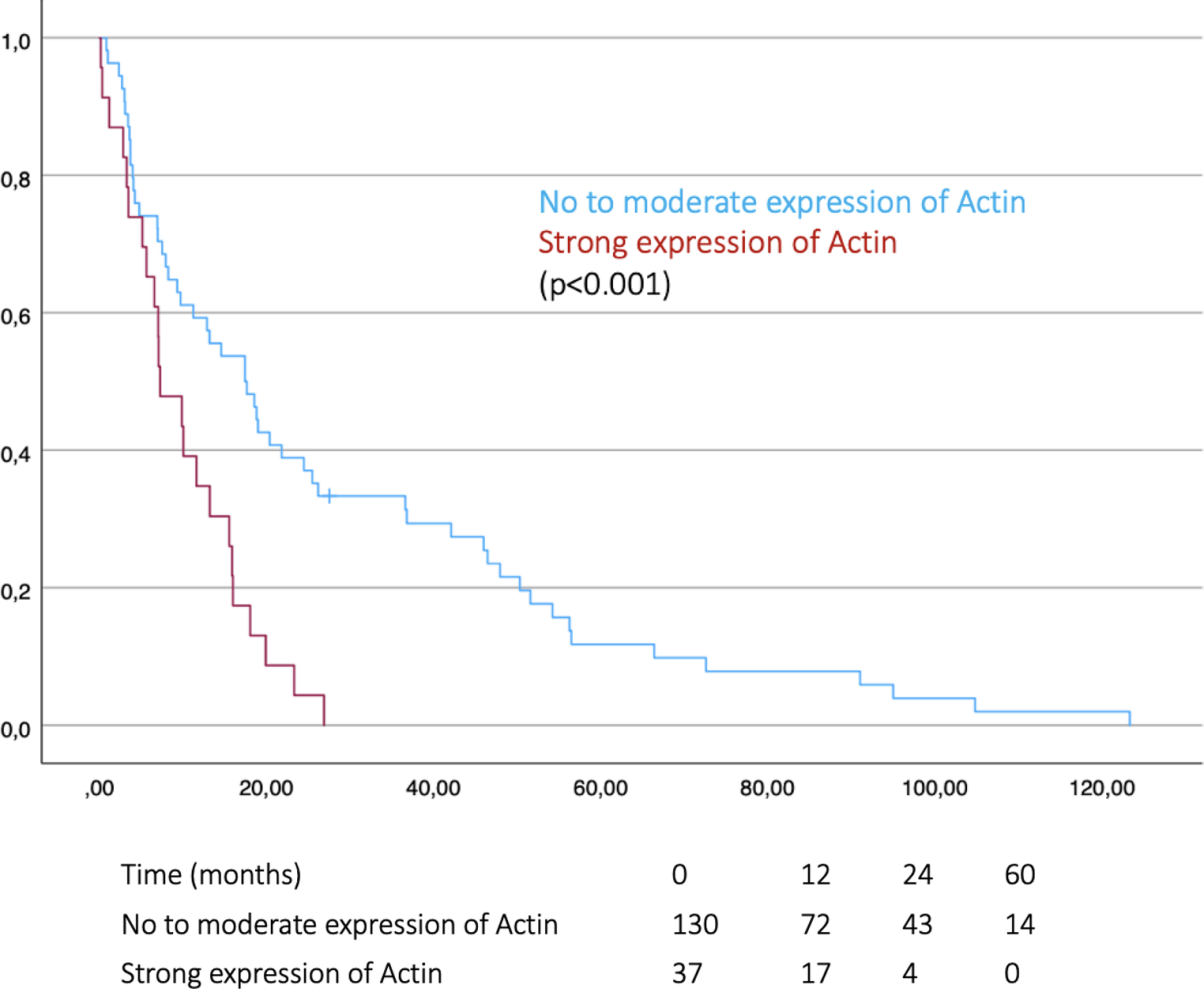
Differences in PFS between patients with strong and patients with no to moderate expression of actin

Strong actin expression was associated with significantly shorter PFS compared to absent or low actin expression (median 7.2 vs. 17.4 months, *p* < 0.001). PFS development in accordance with actin expression is illustrated in Fig. 4. These findings remained consistent in subgroup analyses stratified by IDH status. In contrast, PFS was not significantly influenced by Cx43 or GAP-43 expression. GAP-43 expression was not significantly associated with either PFS or OS in Kaplan–Meier analyses or multivariable Cox regression models (all *p* > 0.05).

**Fig. 5:**
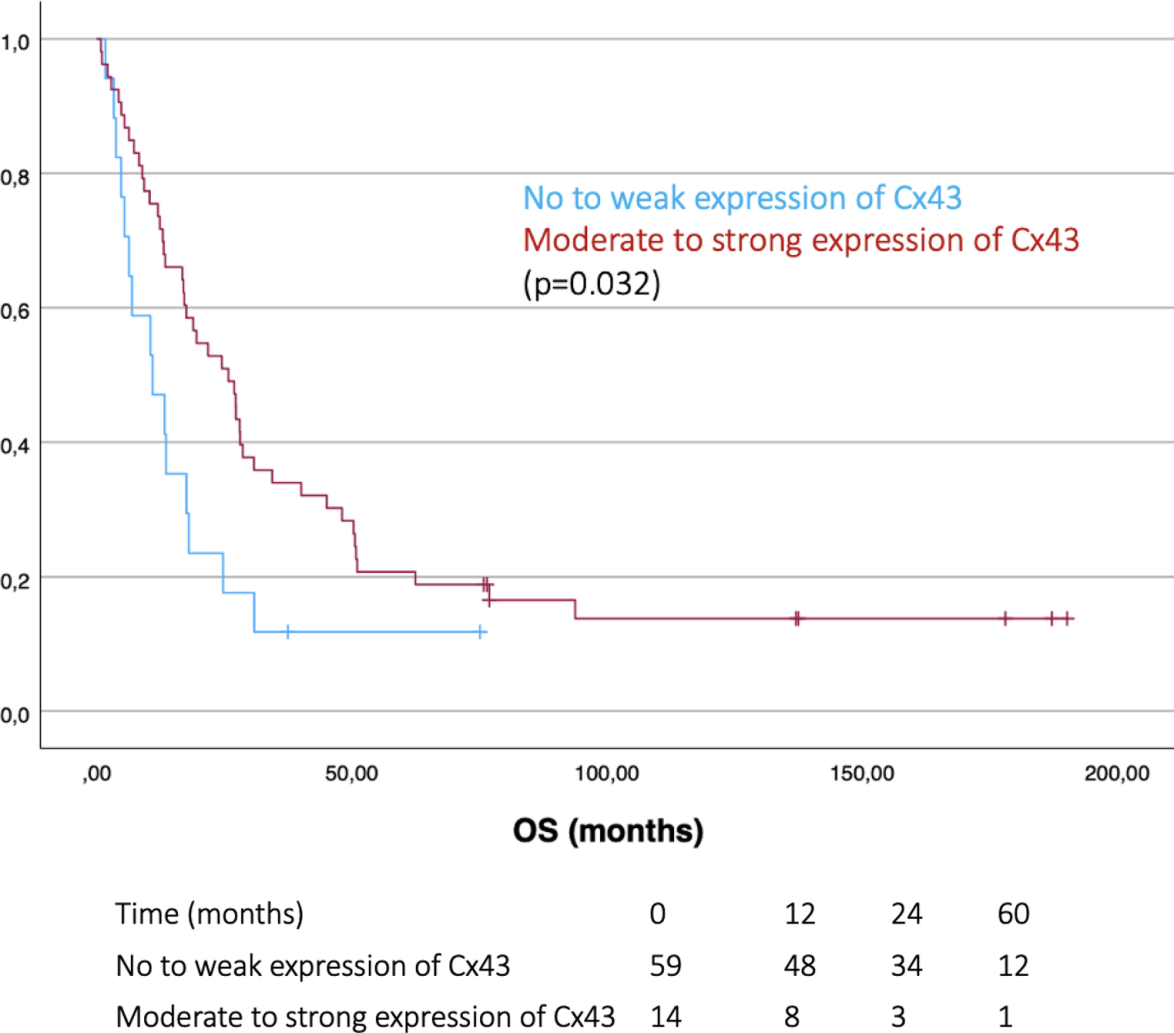
In IDH-wildtype patients, moderate to strong expression of Cx43 led to a significantly more favorable OS (*p* = 0.032)

In the overall cohort, Cx43 expression did not have a significant impact on OS (*p* > 0.05). Only IDH-wildtype patients with higher Cx43 expression demonstrated significantly prolonged OS, as illustrated in Fig. 5 (*p* = 0.032).

**Fig. 6:**
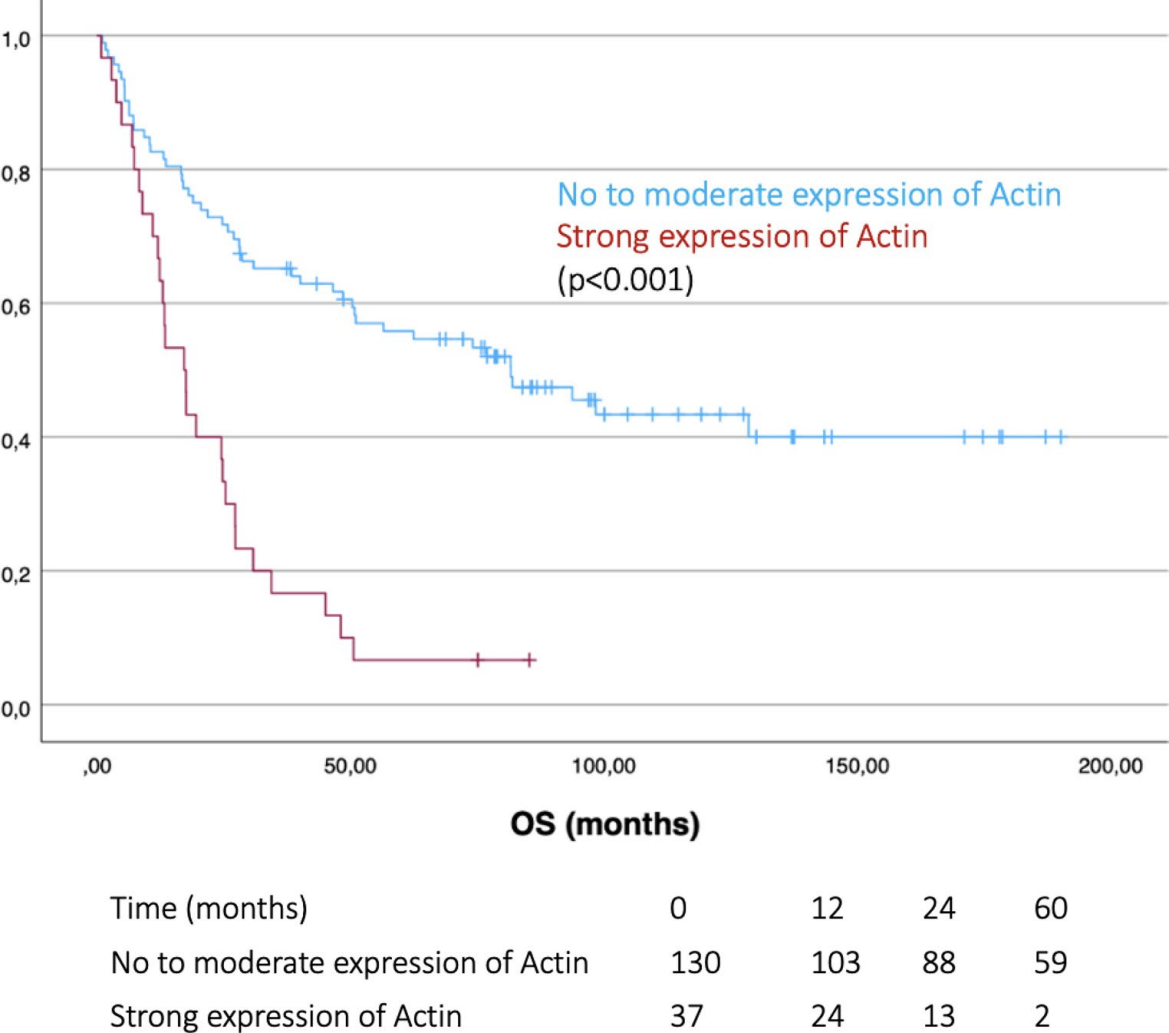
Strong actin expression was significantly associated with reduced OS compared to tumors with absent or lower actin expression (*p* < 0.001)

Patients with strong actin expression showed significantly reduced OS (median 17.1 vs. 81.5 months, *p* < 0.001). These findings remained consistent in subgroup analyses for IDH-wildtype patients (*p* = 0.016). The relevant OS data are illustrated as Kaplan-Meier curves in Fig. 6.

In multivariable Cox regression analyses, both elevated actin expression (HR 3.2, 95% CI 1.8–5.7) and higher Cx43 expression in IDH-wildtype gliomas (HR 0.58, 95% CI 0.35–0.95) remained independently associated with survival outcomes after adjustment for preoperative KPS, extent of resection, and WHO grade (all *p* < 0.05).

## Discussion

In this study, we analyzed the immunohistochemical expression of three pathophysiologically relevant proteins—Cx43, GAP-43, and actin—in a large real-world cohort of patients with de novo diagnosed gliomas, with our clear focus on their clinical therapeutic potential and the emerging role of cell–cell communication as a key driver of future treatment strategies.

We observed, that higher Cx43 expression was associated with improved OS specifically in IDH-wildtype tumors. Furthermore, significantly lower expression levels of actin and Cx43 in IDH-mutant tumors, compared to IDH-wildtype gliomas, underscored the distinct molecular profiles of IDH-defined glioma subtypes. Moreover, our findings demonstrate that elevated actin expression is strongly associated with prognostically unfavorable features like CE in MRI, higher WHO grade, lower patient performance and consequently shorter PFS and OS, highlighting its potential role as a marker of aggressive tumor biology. Finally, incidentally diagnosed tumors showed reduced GAP-43 expression, potentially respecting less active tumor phenotype at the time of diagnosis. Thus, our data provide clinically relevant evidence linking cytoskeletal and connectivity-associated proteins to survival and tumor behavior in glioma patients.

Importantly, these findings position cell–cell communication as a central driver of next-generation therapies, with the potential to redefine how we approach treatment in the near future.

Recent preclinical studies have demonstrated that pharmacological inhibition of Cx43-associated signaling pathways can exert antitumor effects and improve survival in experimental brain tumor models, suggesting that targeting this pathway could represent a promising strategy for future glioma therapies [31–33].

Gliomas are characterized by pronounced cellular plasticity, infiltrative growth and complex interactions with the surrounding neurons. Beyond established molecular markers such as IDH status, increasing experimental evidence suggests that cytoskeletal dynamics and intercellular communication critically contribute to tumor progression, invasion and treatment resistance [18, 34, 35]. Their regulation may facilitate tumor cell integration into neural networks and promote aggressive biological behavior [18, 19, 21, 34]. Notably, preclinical studies have already suggested potential therapeutic benefits from pharmacological inhibition of these signaling pathways [31, 32]; however, clinical data validating their relevance in real-world patient cohorts remain scarce. Furthermore, while underlying mechanisms have been extensively explored in experimental models, comprehensive clinical data correlating their expression with patient functional status, tumor radiomorphological characteristics and survival outcomes in large real-world groups remain limited. By systematically investigating these proteins in a well-defined cohort of IDH-mutant and IDH-wildtype gliomas, our study bridged the translational gap and provided clinically meaningful insights into how cytoskeletal and connectivity-related pathways may shape tumor burden and patient prognosis.

The baseline characteristics of our cohort were largely consistent with previously published glioma series [36–38]. Age distribution, male predominance and the proportion of eloquent tumor locations were comparable to existing literature [36, 39, 40]. Moreover, the median follow-up period in our cohort was applicably long, allowing for a robust evaluation of long-term clinical outcomes. Likewise, radiological tumor volumes and surgical parameters including high extent of resection were within the expected range for contemporary neuro-oncological practice, supporting the representativeness and validity of our dataset [36–41].

Upregulated Cx43 expression positively influenced OS in IDH-wildtype tumors, whereas no survival benefits were observed in IDH-mutant gliomas. In IDH-wildtype tumors, altered Cx43 expression may therefore modulate tumor progression through effects on cellular connectivity, microenvironmental adaptation or therapy response. In previous studies, Cx43 expression inversely correlated with mitotic activity, consistent with prior reports showing reduced Cx43 levels in highly proliferative glioblastomas and an association with lower mitotic indices. This supports experimental data suggesting that Cx43 may exert tumor-suppressive effects through cell cycle regulation, including G1 delay and modulation of cell cycle-related gene transcription [15]. In contrast, the comparatively indolent biology of IDH-mutant gliomas may diminish the functional impact of Cx43-mediated signaling. These findings indicate that Cx43 does not act as a universal prognostic marker but rather exerts context-dependent effects shaped by molecular tumor subtype.

We observed significantly lower expression levels of actin and Cx43 in IDH-mutant gliomas compared to IDH-wildtype tumors. The increased expression of these proteins in IDH-wildtype tumors may therefore reflect greater structural plasticity, migratory capacity and integration into neural circuits, as previously suggested [22, 24, 42]. Conversely, reduced expression in IDH-mutant tumors may correspond to their comparatively restrained invasive behavior and more stable cellular architecture [10, 43]. Together, these findings suggest that differential regulation of cytoskeletal and connectivity-associated proteins may contribute to the distinct clinical trajectories of IDH-defined glioma subtypes.

Elevated actin expression was strongly associated with prognostic unfavorable tumor features including CE on MRI and advanced WHO grade, lower patient performance and consequently shorter PFS and OS, underscoring its potential role as a marker of aggressive tumor biology. Importantly, these associations remained significant after multivariable adjustment for established prognostic factors, indicating that the observed effects were not solely attributable to differences in tumor grade, functional status, extent of resection, or IDH status. Actin is a central component in glioma cell migration and infiltration into surrounding healthy brain tissue and plays a crucial role in cellular motility and structural adaptation [43, 44]. Increased actin expression may facilitate enhanced migratory capacity of glioma cells, promoting diffuse infiltration into surrounding brain tissue and limiting the effectiveness of local therapies such as surgical resection, which has already been suggested in previous studies [45, 46]. Moreover, dynamic cytoskeletal remodeling has been linked to resistance mechanisms, including adaptation to hypoxic stress and therapeutic pressure [43, 47]. Contrast enhancement reflects areas of blood–brain barrier disruption, where the specific role of actin in mediating vascular permeability and microenvironmental interactions remains incompletely understood. Thus, the association between high actin expression and contrast-enhancing tumor regions further supports its link to a more proliferative and biologically active phenotype. Taken together, these findings suggest that increased actin expression reflects heightened structural plasticity and invasiveness, translating into earlier progression and reduced survival in affected patients.

Emerging preclinical evidence suggests that Cx43-associated signaling pathways may represent potential therapeutic targets in glioma. Experimental studies have demonstrated that modulation of Cx43-related signaling, including inhibition of gap junction-mediated communication and administration of Cx43-derived peptides, can disrupt glioma cellular networks and enhance the efficacy of cytotoxic therapies in preclinical models [31, 48]. In particular, pharmacological modulation of gap junction communication has been shown to sensitize glioblastoma cells to temozolomide-induced cell death, while tonabersat, a blood–brain barrier–penetrating gap junction modulator, has been reported to reduce intercellular communication and enhance temozolomide-mediated cytotoxicity [49]. Furthermore, more recent work has identified tonabersat, a blood–brain barrier–penetrating gap junction inhibitor, as a potential candidate capable of reducing intercellular communication within glioblastoma networks and enhancing temozolomide-mediated cytotoxicity through inhibition of Cx43-dependent signaling [48]. Although these findings are currently limited to experimental studies, they highlight the biological relevance of Cx43-associated pathways. In this context, our clinical observations provide complementary real-world evidence linking Cx43 expression to glioma behavior and support further mechanistic and translational studies investigating the therapeutic potential of these pathways.

Interestingly, incidentally diagnosed gliomas demonstrated significantly lower GAP-43 expression compared to symptomatic tumors. The reduced expression observed in incidental tumors may, therefore, reflect a biologically less active or not invasive phenotype at the time of diagnosis. This finding aligns with the clinical observation that incidental gliomas are often detected at earlier stages and may exhibit slower progression [37, 50]. Consequently, lower GAP-43 expression could represent a molecular correlate of a more indolent tumor biology in this subgroup. However, further experimental and clinical studies are required to better elucidate the mechanistic basis and prognostic implications of this observation.

Importantly, the expression levels of Cx43, GAP-43 and actin did not influence postoperative patient safety. No significant associations were observed between protein expression and the occurrence of surgical complications, including new neurological deficits, postoperative hemorrhage or other procedure-related adverse events. These findings suggest that, although actin and Cx43 were linked to tumor aggressiveness and survival, their expression does not translate into an increased perioperative risk. Thus, the biological impact of these proteins appears to be primarily related to tumor behavior rather than surgical vulnerability or short-term postoperative morbidity.

This study has limitations that should be acknowledged. First, it represents a monocentric analysis, and although our cohort was well characterized and comparable with the existing literature, the findings may not be fully generalizable to other patient populations. External validation in independent, multicenter cohorts is therefore warranted. Second, due to the inclusion period and aiming for long-term follow-up, all patients were initially diagnosed according to the WHO classification of tumors of the central nervous system of 2016. Furthermore, although all tumors were reclassified according to the revised 5th edition of the WHO Classification of Tumors of the Central Nervous System using all available molecular information, complete molecular profiling was not available for every patient. TERT promoter mutation status was unavailable in a proportion of the retrospective cohort, reflecting changes in routine molecular diagnostics over the study period. Consequently, the precision of molecular subclassification according to the 2021 WHO classification may have been limited in some cases. Future studies should incorporate comprehensive molecular profiling in all patients to further validate our findings. Immunohistochemical expression was assessed using a semi-quantitative scoring system based on experienced observer evaluation, which may introduce a degree of subjectivity and limit reproducibility across institutions. Furthermore, patient ethnicity was not routinely documented in our institutional database and was therefore unavailable for analysis, limiting the assessment of potential ethnic differences in protein expression and clinical outcomes. In addition, automated digital image analysis was not available for this retrospective cohort; future studies should combine quantitative digital pathology with comprehensive molecular profiling to further validate these findings. Further experimental and translational studies are required to clarify the biological pathways involved and to explore potential targeted therapeutic strategies.

## Conclusion

In this large monocentric cohort of de novo IDH-mutant and IDH-wildtype gliomas, we demonstrated that elevated actin expression is associated with unfavorable prognostic features, worse patient performance, and consequently reduced PFS and OS, underscoring its association with aggressive tumor biology. Higher Cx43 expression correlated with longer OS specifically in IDH-wildtype tumors, while GAP-43 expression was reduced in incidental gliomas; neither protein was associated with perioperative patient safety. Together, these findings provide clinically relevant evidence linking cytoskeletal and connectivity-associated proteins to tumor behavior and survival, thereby extending experimental observations in a well-characterized clinical cohort. Recent preclinical studies have suggested that modulation of Cx43-mediated signaling may disrupt glioma cellular networks and enhance the efficacy of standard therapies such as temozolomide. Although our study does not provide functional evidence for such therapeutic approaches, our findings support the biological relevance of these pathways and provide a clinical rationale for future mechanistic and translational studies investigating their therapeutic potential.

## Data Availability

The data supporting this study are available from the corresponding author upon reasonable request.
